# Remodeling the Epigenetic Landscape of Cancer—Application Potential of Flavonoids in the Prevention and Treatment of Cancer

**DOI:** 10.3389/fonc.2021.705903

**Published:** 2021-06-21

**Authors:** Weiyi Jiang, Tingting Xia, Cun Liu, Jie Li, Wenfeng Zhang, Changgang Sun

**Affiliations:** ^1^ College of First Clinical Medicine, Shandong University of Traditional Chinese Medicine, Jinan, China; ^2^ Clinical Medical Colleges, Weifang Medical University, Weifang, China; ^3^ Department of Oncology, Weifang Traditional Chinese Hospital, Weifang, China; ^4^ Qingdao Academy of Chinese Medical Sciences, Shandong University of Traditional Chinese Medicine, Qingdao, China

**Keywords:** flavonoids, natural compounds, phytochemicals, epigenetic, epigenome, cancer, HDAC, DNMT

## Abstract

Epigenetics, including DNA methylation, histone modification, and noncoding RNA regulation, are physiological regulatory changes that affect gene expression without modifying the DNA sequence. Although epigenetic disorders are considered a sign of cell carcinogenesis and malignant events that affect tumor progression and drug resistance, in view of the reversible nature of epigenetic modifications, clinicians believe that associated mechanisms can be a key target for cancer prevention and treatment. In contrast, epidemiological and preclinical studies indicated that the epigenome is constantly reprogrammed by intake of natural organic compounds and the environment, suggesting the possibility of utilizing natural compounds to influence epigenetics in cancer therapy. Flavonoids, although not synthesized in the human body, can be consumed daily and are common in medicinal plants, vegetables, fruits, and tea. Recently, numerous reports provided evidence for the regulation of cancer epigenetics by flavonoids. Considering their origin in natural and food sources, few side effects, and remarkable biological activity, the epigenetic antitumor effects of flavonoids warrant further investigation. In this article, we summarized and analyzed the multi-dimensional epigenetic effects of all 6 subtypes of flavonoids (including flavonols, flavones, isoflavones, flavanones, flavanols, and anthocyanidin) in different cancer types. Additionally, our report also provides new insights and a promising direction for future research and development of flavonoids in tumor prevention and treatment *via* epigenetic modification, in order to realize their potential as cancer therapeutic agents.

## Introduction

Cancer is a chronic consumptive disease that has not been overcome yet and has become a global health problem threatening human life (1, 2). Developments in the field of oncology suggest that cancer is regulated not only by genetics but also by epigenetic programming (3). First, epigenetic mechanisms can circumvent changes in the DNA sequence and directly target the expression of proto-oncogenes and tumor suppressor genes to induce carcinogenesis (4, 5). Second, epigenetic abnormalities occur in the early stage of carcinogenesis and precede mutations which also occur in somatic cells (6); therefore, such mechanisms have become a new therapeutic avenue to conquer tumors. More importantly, in the complex and multi-dimensional process of carcinogenesis, these mechanisms, which differ from genetics, are frequently bidirectional and reversible (7). And defects of epigenetics are largely a consequence of lifestyle and habits such as inadequate consumption of natural compounds, smoking, and drinking (8, 9), and they can also reverse abnormal epigenetic effects through proper intake of natural compounds (10, 11). These facts make epigenetics attractive for the development of new treatments.

Flavonoids are phytochemicals that are frequently consumed on a daily basis. Because of their remarkable biological activities, flavonoids have recently attracted scientific interest as they are beneficial to health and reduce the risk of disease (12, 13). In the field of cancer, evidence of cancer treatment effects of flavonoids through epigenetic pathways is continuously being investigated. For example, kaempferol, which occurs in grapefruit, broccoli, and other plants, can regulate the activity of histone deacetylase (HDAC) in various cancers (14). Epigallocatechin-3-gallate (EGCG) in tea can affect expression of DNA methyltransferases, HDACs, and noncoding RNAs in cancer cells (15–17). Key evidence such as this improves our understanding of the importance of diet for cancer prevention and suggests the potential of dietary flavonoids for epigenetic therapy against cancer.

Epigenetic mechanisms of cancer have been described in detail in previous studies (18, 19). Thus, we review the core mechanism of epigenetics and abnormal regulation in cancer to provide a clear groundwork. Recent research progress regarding anticancer efficacy of all types of flavonoids targeting epigenetic regulation pathways is reviewed here, and their potential for cancer prevention and treatment is elaborated to provide evidence for the anticancer effects of food and improve the understanding of respective epigenetic mechanisms to support the development of new treatment strategies and chemoprophylaxes.

## Epigenetics and Cancer

Epigenetics are heritable changes in gene expression without alterations in DNA sequences. There are numerous such phenomena, including DNA methylation, histone modification, genomic imprinting, maternal effects, and RNA editing (20). However, regarding physiological and pathological gene regulation of mammals, epigenetic effects predominantly refer to three aspects, i.e., DNA methylation, histone modification, and noncoding RNA regulation (21). Flavonoids also regulate cancer epigenetic abnormalities through these three aspects.

### DNA Methylation

In the field of epigenetics, DNA methylation regulation is the most studied mechanism which can directly modify gene expression without changing genetic information and participates in various biological processes such as genomic stability, regulation of gene transcription, embryonic development, and tumorigenesis (22–24). DNA methylation modifications are common in human genomic DNA, covalently binding methyl groups to the fifth carbon in the cytosine group of CpG dinucleotides to form 5-methylcytosine. CpG dinucleotides are distributed inhomogeneously in the human genome and are more abundant in promoter regions of genes (25). Gene regions with a higher proportion of CpG are termed CpG islands and occur in promoters of more than 60% of genes (26). Hypermethylation of a promoter CpG island can inactivate the gene, whereas promoter CpG islands of transcriptionally active DNA sequences are typically not methylated (18). For example, even though the vast majority of CpG islands in developmental and differentiated tissues remain methylated, a small number of CpG islands are methylated in a tissue-specific manner to inhibit gene expression (27). In a few cases, DNA hypermethylation can lead to abnormal gene activation. This abnormal regulation has been described in detail in other reports (28), however, in-depth elaboration of this process is beyond the scope of this review.

**Figure 1:**
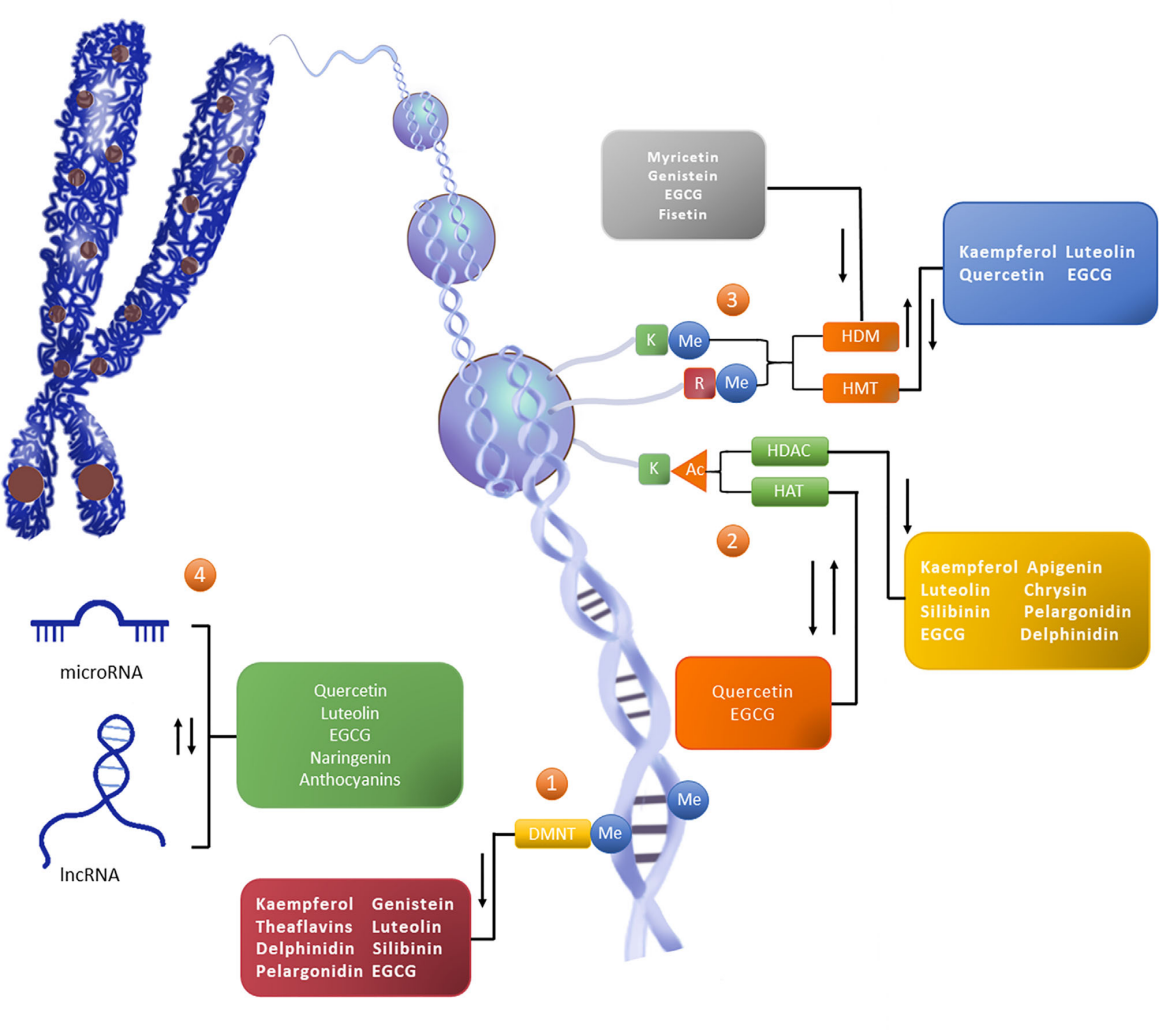
Flavonoids reshape the epigenetic landscape of cancer. Flavonoids exert epigenetic anticancer activity by regulating the level of DNA methylation, histone modification, and the expression of noncoding RNA. The addition of epigenetic markers depends on writers (such as DNMT, HMT, and HAT), except for erasers (such as HDAC and HDM) which depend on readers (not marked in the figure). ①The level of CpG island methylation (Me) in the promoter region of the target gene is increased under the catalysis of DNMT, thus downregulating the activity of the gene. ②Histone modification occurs on lysine (K) and arginine (R) residues in the histone tail, in which K is the site of histone acetylation (Ac) modification. HAT promotes gene expression, while HDAC can reduce the level of histone acetylation to reduce gene activity. ③Histone methylation can occur in K and R. HMT and HDM catalyze epigenetic regulation, and their effects on gene activity vary with amino acid sites and methylation patterns. ④Noncoding RNAs are also an important part of epigenetic networks. Noncoding RNAs, represented by miRNA and lncRNA, can interact with DNA, mRNA, and proteins, and participate in gene expression processes such as chromosome modification, transcriptional interference, posttranscriptional modification, and translation regulation. In general, flavonoids can play the role of DNMTi, HDACi and HDMi, and can promote or inhibit the activity or expression of HAT,HMT and ncRNA.

DNA methylation requires catalysis by DNA methyltransferases (DNMTs) (29). Five types of DNMTs including DNMT1, DNMT3a, DNMT3b, DNMT2, and DNMT3L have been identified (30). The first three types are considered to exert methyltransferase activity: DNMT1 is mainly responsible for maintenance methylation (i.e., identifying the DNA strand that has been methylated and then methylating the complementary strand), while Dnmt3a and Dnmt3b are involved in *de novo* methylation to methylate unmethylated DNA double-strands (31). Abnormal DNA methylation leads to epigenetic imbalance which is an important mechanism underlying tumorigenesis (32). Compared with somatic cells, cancer cells are hypermethylated in the promoter regions of multiple cancer suppressor genes (e.g. MGMT, CdH1, E-cadherin, BRCA1) which is considered a common regulator of transcriptional silencing of tumor suppressor genes, while hypomethylation is observed in the whole genome of cancer cells and is associated with upregulated expression of proto-oncogenes (such as cyclinD2, PAX2, PAX2, and ABCB1) (33, 34). Most flavonoids exert strong effects on DNA methylation (**Figure 1**), and hesperidin and naringenin are candidate drugs that inhibit DNMTs.

### Histone Modification

In addition to DNA methylation which occurs directly on gene sequences, the chromatin structure is also affected by histone modification which is associated with DNA replication, transcription, and repair through histone-DNA or histone-histone interaction (35). Histones are basic proteins of eukaryotes that bind to chromosomal DNA. A histone octamer formed by two molecules of H2A and H2B and two molecules of H3 and H4, together with 147 base pairs of DNA form nucleosomes which participate in the formation of chromosomes (36). Histones in nucleosomes can undergo a variety of epigenetic regulation through the free N-terminal. According to the different modes of action, histone modification can be assigned to methylation, acetylation, phosphorylation, adenylation, ubiquitin, and ADP ribosylation (37). Acetylation and methylation are well-studied regulation modes (36, 38), which are also the main histone modifications mode of natural anti-tumor drug flavonoids (**Figure 1**).

#### Histone Acetylation

Histone acetylation is the best characterized histone modification and plays an important role in gene regulation, chromatin structure, and tumorigenesis (39–41). It is generally accepted that high acetylation activates and low acetylation inhibits gene expression (42–44). Histone acetylation is frequently modified on N-terminal lysine sites of H3 and H4 and is regulated by histone acetyltransferase (HAT) and HDAC (18, 45). There are three HAT families, i.e., the Gcn5-related N-acetyltransferase (GNAT) family which occurs in the nucleus and is associated with transcription; the MYST family, mainly comprising Ybf2-Sas3, Sas2, MOZ, and Tip60, which occur in the cytoplasm and participate in posttranslational modification; and the p300/cbp family which is responsible for transcription and posttranslational modification (46, 47). Based on structural characteristics and catalysis, HDACs are assigned to the following four categories – class I: HDAC1, 2, 3, and 8; class II: HDAC4, 5, 6, 7, 9, and 10; class III: sirtuins (SIRT1-7); and class IV: HDAC11. HDACs such as those of classes I, II, and IV show structural homology and require zinc ions to activate their catalytic effects, while sirtuins require NAD+ as a cofactor to exert catalysis (48).

Histone acetylation plays an important role in the development of cancer. HDAC and HAT can interact with proto-oncogenes and tumor suppressor genes and thereby interfere with the regulation of these genes during tumor cell proliferation, metastasis, and apoptosis (49, 50). For example, during promyelocytic leukemia, the mSin3-HDAC complex can deacetylate key genes and block transcription, leading to tumorigenesis. An imbalance in HAT activity can also lead to cancer. For example, p300/cbp can be reduced by the binding of viral oncoprotein (E1A), indicating the relationship between HAT and tumorigenesis.

#### Histone Methylation

Methylation is also involved in epigenetic regulation of histones. The N-terminal lysine and arginine residues of H3 and H4 are reversibly modified through catalysis of histone methyl invertase (HMT) and histone demethylase (HDM) (51). Histone methylation regulates the silencing and activation of gene transcription, and the specific biological behavior depends on the amino acid residue sites (different parts of lysine or arginine) and on the form of methylation (monomethylation, dimethylation, or trimethylation) (52). For example, methylation at lysine 9 (H3K9) of histone H3 leads to transcriptional inhibition, whereas trimethylation at lysine K4 (H3K4) and at K9 (H3K9) of histone H3 is responsible for transcriptional activation (53, 54). Imbalances in histone methylation and demethylation are closely associated with cancer. For example, MLL1 can cause H3K4 methylation, leading to acute lymphoblastic leukemia (55); LSD1 is both a classical oncogene and an active lysine demethylase(KDM) that removes the methyl group from H3K4 and H3K9 sites and is considered a therapeutic target for acute leukemia cases (56).

### Regulation of Noncoding RNA

#### miRNA

The current understanding of miRNAs suggests that they are important epigenetic vectors and key factors in anticancer-targeting regulation by flavonoids. miRNAs, which comprise approximately 22 nucleotides, are single-stranded RNAs that are responsible for posttranscriptional silencing of target genes, thereby regulating cell proliferation, differentiation, migration, and other biological processes. As effectors of negative regulation of gene transcription, miRNAs participate in the expression of genetic information in two ways; first, perfect pairing of miRNA transcriptional sequences genes leads to degradation of the target mRNA and interrupts mRNA translation; second, miRNAs may inhibit the transcription process by binding to the incomplete complementary site of the 3’-untranslated region of mRNA. miRNAs can regulate 1/3 human gene and form a complex regulatory network with target genes. A given miRNA may correspond to multiple target genes, and a target gene may also be regulated by multiple miRNAs (57–59).

There is increasing evidence for the relationship between miRNAs and cancer. miRNA expression profiles can be used to distinguish normal tissue from cancerous tissue and to classify different types of cancer (60, 61). In addition, miRNAs play a role in carcinogenic and tumor-inhibitory signaling pathways, e.g., miRNAs of the miR-34 family act as a signal regulator in the p53 pathway, and miR-16 negatively regulates mitogenic signals and blocks the cell cycle. Interestingly, regulation of miRNAs in cancer is also affected by DNA methylation and histone modification, as discussed above. For example, hypermethylation of miR-9-1 was observed in breast cancer, and the MIR-17-92 cluster in hepatocellular carcinoma may be regulated by HDAC inhibitors (62, 63). Taken together, epigenetics is not an isolated complex of processes but a multilevel interrelated regulatory network, and miRNAs play an indispensable role in this regulatory mechanism.

#### lncRNA

Unlike miRNA, lncRNA, which is noncoding RNA exceeding 200 bp in length, is well-known for its regulatory effects on the cell cycle, cell differentiation, and epigenetic effects on biological processes (64, 65). Some recent studies examined epigenetic effects of lncRNAs. Their long chain structures enable lncRNAs to form complex secondary and tertiary structures which can bind to DNA, RNA, or proteins, reshape chromatin structures, and participate in transcriptional and posttranscriptional regulation (66–68). Although the specific functions of lncRNAs remain to be elucidated, their unregulated expression patterns have been shown to be related to diseases such as cancer. This type of long-chain RNA participates in malignant mechanisms such as proliferation, migration, and drug resistance and is of clinical importance regarding early diagnosis and prognosis of tumors (69). For example, lncRNA-p21 can induce apoptosis. Highly upregulated in liver cancer is considered a potential biomarker for breast cancer with liver metastasis. High expression of HOX antisense intergenic RNA in breast cancer is associated with invasiveness. lncRNAs have become one of the core targets of tumor research as they may be highly informative regarding cancer. lncRNA therapy can be an adequate tumor treatment option, and dietary phytochemicals such as flavonoids have been examined in the past few years to assess their therapeutic potential to regulate lncRNAs (**Figure 1**).

## Epigenetic Effects of Flavonoids During Cancer

Cancer is affected by interactions of genetics and epigenetics (1). Compared with the genome, the epigenome is often affected by acquired factors such as environment and diet (70, 71). To date, more than 4,000 flavonoids have been identified in plants, and these compounds are common in vegetables, fruits, and tea. The role of flavonoids in the treatment of diseases has been studied extensively (72–74). Prophylactic flavonoids can chemically reverse adverse epigenetic marking in cancer cells, which may inspire researchers to explore novel avenues of cancer prevention and early treatment (73).

Flavonoids, the largest group of secondary metabolites in the polyphenol family, are produced by plants where they provide flavor, color, and health effects (75, 76), and they have attracted research attention because of their effective antioxidant effects which may prevent environmental damage (73). **Table 1** shows different subtypes of flavonoids from dietary and medicinal plant sources. Flavonoids exert various pharmacological activities in mammals and have been commonly used in studies on various diseases (99, 100). In vivo and *in vitro* studies showed that these plant compounds exert beneficial effects such as antitumor, antibacterial, antiviral, liver-protective, anti-inflammatory, antipyretic, analgesic, antihypertensive, and anti-aging effects, in addition to neutralization of free radicals, improvement of immune functions, and beneficial effects on the cardiovascular system (101, 102).

**Table 1:** Dietary and plant sources of the different types of flavonoids.

Flavonoid class	Compound name	Dietary sources	Reference
Flavones	Apigenin	Achillea millefolium L	(77)
	Luteolin	Chrysanthemi Flos	(78)
	Tangeretin	Citrus Reticulata	(79)
	Diosmin	Black cardamom	(80)
	Chrysin	Propolis	(81)
Isoflavones	Daidzein	Soybeans	(82)
	Daidzein	Cyathulae Radix	(83)
	Daidzein	Sojae Semen Praeparatum	
	Genistein	Miso	(84)
	Genistein	Eucommiae Cortex	(83)
Flavonols	Kaempferol	Radix Bupleuri	(83)
	Kaempferol	Polygoni Avicularis Herba	(85)
	Kaempferol	Hippophae Fructus	(86)
	Kaempferol	Paeoniae Radix Alba	(83)
	Myricetin	Mori Fructus	(87)
	Myricetin	Hippophae Fructus	(88)
	Myricetin	Ginkgo Folium	(89)
	Myricetin	Red wine	(90)
	Myricetin	Black tea	(91)
	Quercetin	Hedyotis Diffusae Herba	(83)
	Quercetin	Radix Bupleuri	
	Quercetin	Toosendan Fructus	
	Quercetin	Slicing beans	(92)
	Quercetin	Broccoli	(93)
Flavanones	Naringenin	Grapefruit pulp	(94)
	Naringenin	Prunus yedoensis Mats	(95, 96)
	Hesperidin	Viticis Fructus	
	Hesperidin	Asteris Radix Et Rhizoma	
	Naringin	Grapefruit juice	
Flavanols	EGCG	Green tea	(97)
	EGCG	Ginkgo Semen	
	ECG	Black tea	
	EGC	Green tea	
Anthocyanidins	Delphinidin	blueberry	(98)
	Delphinidin	Ephedra Herba	(83)
	Pelargonidin	Fritillariae Thunbrgii Bulbus	
	Pelargonidin	Hippophae Fructus	
	Pelargonidin	Mori Cortex	

**Figure 2:**
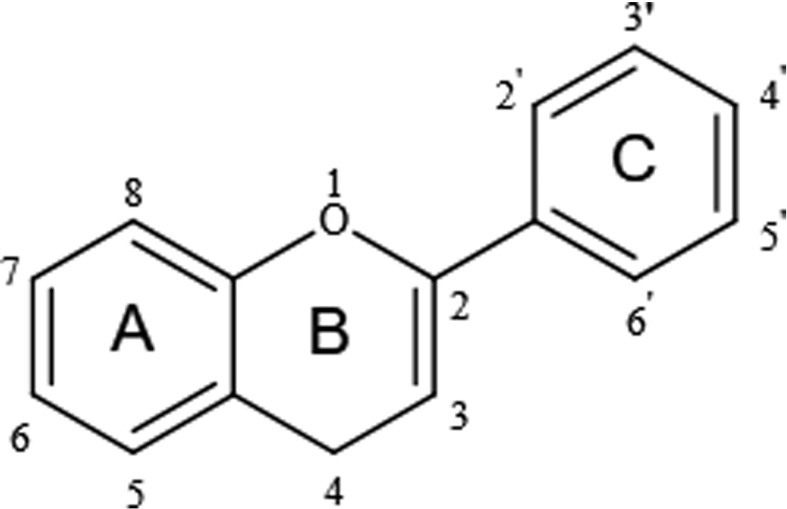
Basic structure of flavonoids.

**Figure 3:**
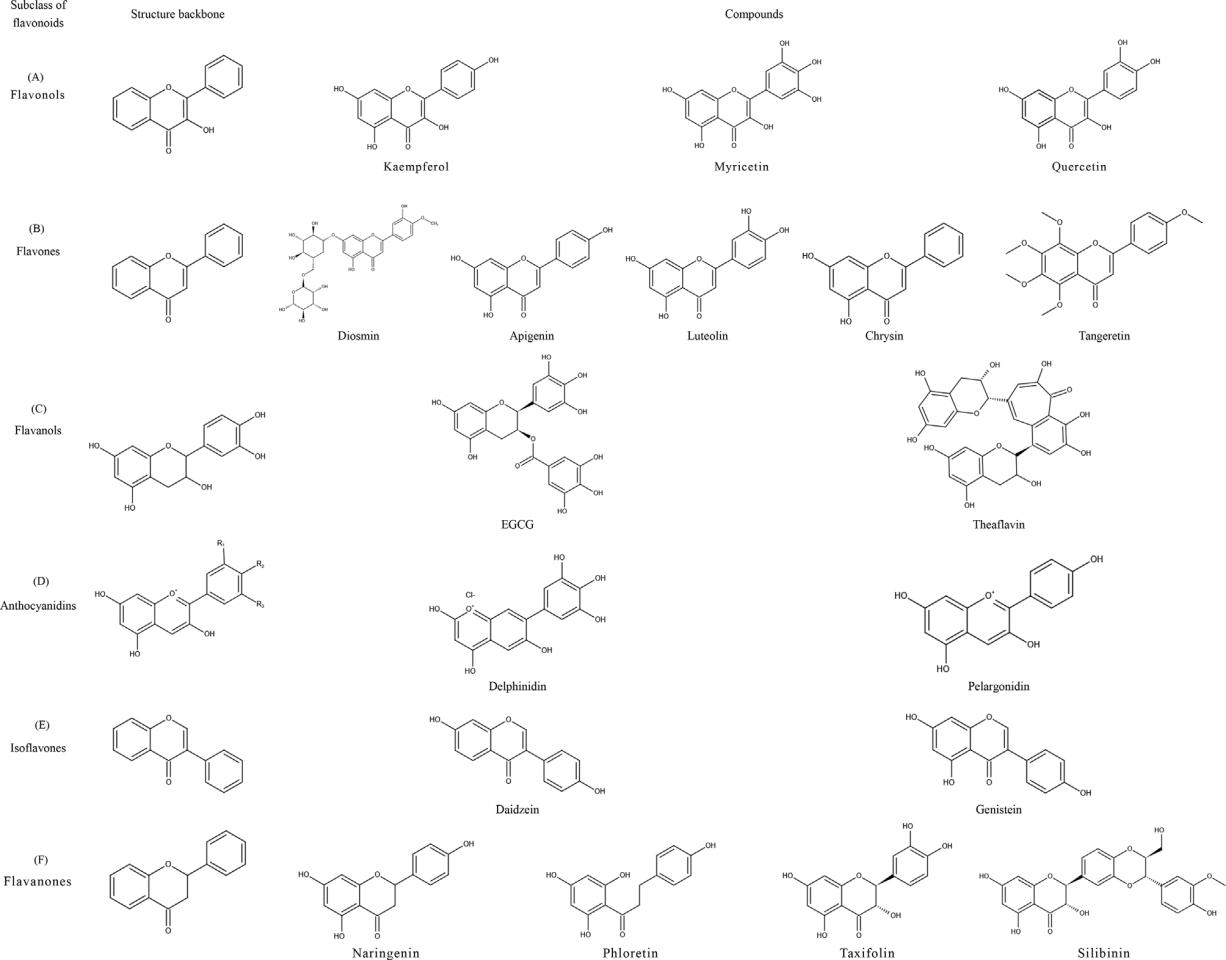
Structural characteristics and representative compounds of six flavonoids subclasses. C6-C3-C6 shows the core structure of flavonoids with twobenzene rings linked to each other by three carbon atoms. After different modifications, flavonoids can occur in six subclasses with different characteristics: flavonols, flavones, isoflavones, flavanones, flavanols, and anthocyanidins; structures are shown in the figure. **(A)** Flavonols: contain 3-hydroxyl flavone analogs. **(B)** Flavones: the ketone of C4 in the C-ring is connected with C2-C3 by a double bond. **(C)** Flavanols: 3-hydroxy derivatives of flavanones; the hydroxyl is located at the third position of the C-ring. **(D)** Anthocyanidins: glavylium cation binding hydroxyl and/or methoxy groups at the R, R, and R sites. **(E)** Isoflavones: the B-ring is connected at C3 of the C-ring instead of C2. **(F)** Flavanones: contain flavone analogs with a C2-C3 single bond structure.

Flavonoids share a particular molecular structure which is formed by the connection of two benzene rings with three carbon atoms, by which these compounds differ from other plant compounds (99, 100) (**Figure 2**). Flavonoids are assigned to six subcategories based on their structures (103). **Figure 3** describes the structural characteristics and representative compounds of the six flavonoid subgroups in detail. In the following sections, we revisit epigenetic cancer effects of flavonoids including flavonols (kaempferol, myricetin, and quercetin), flavones (diosmin, apigenin, luteolin, and chrysin), isoflavones (daidzein and genistein), flavanones (naringenin, naringin, hesperidin, phlorizin, taxifolin, and silibinin), flavanols (EGCG and theaflavins), and anthocyanidins (delphinidin and pelargonidin).

### Flavonols

#### Kaempferol

Kaempferol, a flavonol compound, is derived from Zingiberaceae plants and commonly occurs in Ardisiae Japonicae Herba, Dysosmae Verspiellis Rhixoma Et Radix, Lobeliae Chinensis Herba, strawberries, grapefruit, grapes, broccoli, and other vegetables, fruits, and teas. Its physiological functions include antioxidation, prevention of cancer, inhibition of fat formation, protection of the nervous system and the heart, and antidiabetic and anti-allergy effects (104–106). Recent studies suggested that kaempferol intake is associated with reduced cancer incidence. In a study of epithelial ovarian cancer, the population with the highest intake of kaempferol had a 40% reduction in the incidence of ovarian cancer compared with the lowest fifth (107). In a cohort study, total flavonol intake was associated with reduced risk of pancreatic cancer, with kaempferol showing the highest reduction in cancer risk. In addition, total flavonol intake and kaempferol intake were negatively correlated with the risk of pancreatic cancer in smokers (108). Epidemiological investigations such as these demonstrated anticancer effects of kaempferol.

Part of the antitumor effect of kaempferol is due to its strong epigenetic effects such as targeting histone acetylation. In a previous study by Kim et al., kaempferol exerted the effect of an HDAC inhibitor (HDACi), inhibited expression of G9a, blocked the HDAC/G9a axis, and induced autophagy in gastric cancer cells (14). A different study also reported significant inhibitory effects of kaempferol on HDAC and found that kaempferol hyperacetylated histone complex H3 and inhibited the proliferation and survival of cancer cells in HCT-116 colon cancer cells and human hepatocellular carcinoma cell lines HepG2 and Hep3B (109).

Kaempferol is also thought to regulate DNA methylation. In HCT116, HT29, and YB5 colorectal cancer cell lines, kaempferol was found to bind to DNMT1, and it significantly downregulated DACT2 methylation, upregulated expression of this tumor suppressor gene, blocked the Wnt/β-catenin signaling pathway, and inhibited tumor cell proliferation and migration. This study also found that DACT2, regulated by kaempferol demethylation, reduced tumor load to some extent, indicating the potential of this compound for cancer therapy (110).

#### Myricetin

Exploring flavonols, numerous studies have shown that myricetin derived from myricanagi, Mori Fructus, ippophae Fructus, Ginkgo Folium, etc. has excellent nutritional value and biological activity, and plays a beneficial role regarding antitumor activity, prevention of cardiovascular diseases, lowering blood pressure, lowering blood lipid levels, and reducing complications associated with diabetes and bacteriostasis (111–114). In recent years, with the rise of epigenetic therapy, an increasing number of epigenetic effects of myricetin has been revealed.

Lee et al. observed that several bioflavonoids can inhibit epigenetic regulation of SssI DNMT and DNMT1 in a concentration-dependent manner and reduce the level of DNA methylation. Compared with fisetin and quercetin, myricetin has a more prominent inhibitory ability on the two enzymes. Myricetin (20 μM) inhibited SssI DNMT-related methylation by up to 60%. When mediated by COMT, the IC_50_ value of myricetin inhibiting methylation of DNMT1 was 1.2 μM (115). This study reflects the epigenetic effects of flavonoids such as myricetin, particularly regarding inhibitory effects on DNMT.

Furthermore, epigenetic effects of myricetin nanoparticles have been reported. Silver nanoparticles(AgNPs) made from myricetin can promote oxidative stress responses and DNA damage and induce apoptosis in NIH3T3 mouse embryonic fibroblasts. Subsequent gene ontology analysis revealed that AgNPs promote nucleosome assembly and DNA methylation in the process of inducing apoptosis. A heat map showed that histones hist2h2ac and hist1h2ah of the necroptosis pathway were significantly upregulated after treatment with AgNPs (116). This study showed that this new material plays an important role in the epigenetic regulation of apoptosis, especially at DNA-methylation and histone levels.

JMJD3, a member of the Jmj-C HDM family, is considered a key factor in inflammation and tumorigenesis. Numerous recent studies showed that myricetin exerts significant inhibitory effects on JMJD3. Mallik et al. screened eight compounds, including ZINC15271426, from 65 myricetin analogs in the ZINC database using the molecular model method. They observed excellent docking affinity (all exceeding 9.0 kcal/mol), showing promising application prospects of these compounds as JMJD3 inhibitors (117). In addition, myricetin indirectly regulates deacetylation. Myricetin can activate HDAC SIRT1 to promote HIF-1α expression and inhibit cMyc and β-catenin (118).

#### Quercetin

Quercetin occurs in Hedyotis Diffusae Herba, Radix Bupleuri, Toosendan Fructus, onions, apples, hawthorn, etc. and naturally occurs in the form of glycosides. It is a commonly used natural flavonol which can neutralize free radicals and directly inhibits tumors; furthermore, it exerts biological activities such as anti-inflammatory, antibacterial, antiviral, immune-regulating, neuroprotective, anti-hypertensive, and hypolipidemic effects (119–121). Recent studies showed that quercetin modifies tumor epigenetics and can enhance the effects of epigenetic treatments when administered in combination with other drugs.

During synergistic action of quercetin and curcumin, prostate cancer cell lines PC3 and DU145 showed decreased DNMT activity and increased AR expression which induced apoptosis through the mitochondrial pathway. Compared with the single-drug group, combined administration was more effective and led to increased sensitivity of androgen-resistant prostate cancer cells to androgen receptor-induced apoptosis (122), suggesting the potential of quercetin and curcumin administration for prevention and treatment of prostate cancer.

Flavonoids quercetin and sodium butyrate can change histone acetylation modifications in cancer. Zheng et al. reported that curcumin and sodium butyrate can inhibit tumor cell growth in a dose-dependent manner. In the human esophageal cancer cell line Eca9706, both drugs can restrict cell growth, and combined administration produced stronger inhibitory effects on the proliferation of tumor cells. Western blotting showed that expression of NF-κBp65, HDAC1, and cyclin D1 was downregulated, whereas that of caspase-3 and p16INK4α was upregulated in treatments with quercetin, sodium butyrate, or a combination thereof. The authors also observed a trend of downregulation of HDAC1-IR and upregulation in E-cadherin-IR after combined treatment (123). This study suggests that quercetin and B, particularly in combination, can regulate abnormal epigenetic changes through the HDAC1-IR-NF-κB signaling axis and inhibit growth of the human esophageal cancer cell line Eca9706.

Wang et al. conducted a study on increased antitumor effects of quercetin due to combined administration with arctigenin. Quercetin and/or arctigenin were used to treat prostate cancer cell lines (androgen-dependent LAPC-4 and LNCaP), and compared with the controls and single-drug treatments, combined administration enhanced the pharmacological effects on anticancer cell proliferation. Combined administration of the two drugs also inhibited expression of AR and PI3K/Akt signaling pathways and that of various cancer-related miRNAs such as miR-21, miR-19b, and miR-148a, and it exerted stronger inhibitory effects on tumor cell migration than single-drug treatments (124). The combination of these two natural compounds may provide a new approach for the treatment and prevention of prostate cancer.

Using cell experiments and xenograft models, Pham et al. found that combination of quercetin with the epigenetic drug BET inhibitor JQ1 can enhance anticancer effects on pancreatic and thyroid cancer by inhibiting tumor cell proliferation and inhibit sphere-formation ability of tumor cells, which shows the effect of targeted therapy on tumor stem cells. In addition, knocking out HnRNPA1 can enhance the inhibiting effect of JQ1 on expression of survivin and the sphere-forming ability of cancer cells, which is consistent with the effects of quercetin (125). This shows that quercetin enhances the effects of BET inhibitor JQ1 by inhibiting expression of the hnRNPA1 gene, and combined administration may have promising application prospects for cancer patients, especially regarding patients with thyroid and pancreatic cancer.

Quercetin also showed broad-spectrum epigenetic activity when used in single-drug treatments in which miRNA regulation was most prominent, as confirmed in various forms of cancer. When carcinogenicity of hexavalent chromium Cr(VI) was reduced, long-term administration of BEAS-2B human bronchial epithelial cells Cr(VI) upregulated miRNA-21, downregulated programmed cell death 4 (PDCD4), promoted ROS expression, and induced cell malignancy. This process can be inhibited by quercetin. In a nude mouse model, the tumor incidence in nude mice injected with Cr(VI) + quercetin BEAS-2B cells was significantly lower than that in the Cr(VI) group. Furthermore, after collecting tumors from nude mice and administering quercetin, promotion of miR-21 was associated with inhibition of PDCD4 (126). These results suggest that quercetin can regulate the miRNA-21-PDCD4 axis to inhibit malignant transformation of human bronchial epithelial cells induced by Cr(VI).

Moreover, quercetin inhibits the self-renewal and proliferation of AsPC1 human pancreatic cancer cells by regulating a variety of miRNAs. After cells were treated with quercetin for 12 h, changes in miRNA expression associated with Notch signaling and cell fate were analyzed, and 11 types of miRNAs including let-7c and miR-200b-3p were observed. miR-200b-3p was further examined and was shown to be a key factor controlling cell fate. Subsequent *in vitro* experiments showed that miR-200b-3p enhanced by quercetin inhibited the Notch signaling pathway, adjusted the transformation of the CSC division mode from symmetry to asymmetry, inhibited self-renewal and proliferation of CSCs, promoted cell differentiation, and reduced invasiveness (127).

Anticancer mechanisms of miRNAs regulated by quercetin have also been reported in hepatocellular carcinoma. In HepG2 hepatoma cells, quercetin promoted expression of miRNA-34a and mediated the p53/miR-34a/SIRT1 signaling mechanism to transmit apoptosis signals of cancer cells (128).

Quercetin plays a key role in inhibiting invasion and proliferation of breast cancer cells. The mechanism underlying the regulation of cancer cells is to promote expression of miR-146a, activate caspase-3 and Bax, and induce apoptosis through a mitochondrial-dependent pathway in human breast cancer cell lines MCF-7 and MDA-MB-231 to inhibit proliferation of tumor cells, inhibit expression of EGFR, and reduce invasiveness of tumor cells (129).

In osteosarcoma studies, quercetin enhanced the efficacy of cisplatin by regulating the miR-217-KRAS signaling axis. When human osteosarcoma 143 B cells were treated with quercetin and/or cisplatin, expression of KRAS was downregulated and miR-217 was upregulated, and a combined treatment significantly increased the expression of miRNA-217 (130). This also confirms previous reports which suggested that miR-217 targets KRAS in lung cancer cells to reverse cisplatin resistance (131).

### Flavones

#### Diosmin

Diosmin is a well-known natural flavonoid used for the treatment of chronic venous insufficiency and varicose veins. Numerous recent studies showed that diosmin exerts various pharmacological activities including anti-inflammatory, antioxidative, antidiabetic, anticancer, antimicrobial, liver-protective, neuroprotective, cardiovascular-protective, kidney-protective, and retina-protective effects (132, 133).

Diosmin is effective against tumors on a genetic and epigenetic level. Epigenetic intervention effects of three flavonoids and their genotoxicity and apoptosis-inducing effects were assessed in DU145 prostate cancer cells. Naringin, diosmin, and hesperidin reduced the number of cancer cells and proliferation to varying degrees, and diosmin showed the strongest gene toxicity and considerable proapoptotic activity. This study also assessed changes in global DNA methylation, and diosmin downregulated the level of 5-methyl-20-deoxycytidine in DU145 cell lines and mediated DNA demethylation to regulate the epigenome of tumor cells (134).

#### Apigenin

Apigenin, which is common in medicinal plants, vegetables and fruits, including Menthae Herba, Radix Salviae, Codonopsis Radix, Chrysanthemi Flos, celery, onions, and oranges, is a kind of natural flavonoids with various pharmacological activities (135–137). It has been used in studies on many diseases because of its low toxicity and strong biological effects, and it has been reported to exert substantial anti-inflammation, antioxidation, antidiabetic, antitumor, antibacterial, and antiparasitic effects. Moreover, this compound can improve the symptoms of chronic diseases, such as depression, insomnia, amnesia, and Alzheimer’s disease (138–140).

Tseng et al. reported that apigenin downregulated the expression of cyclins A, B, and CDK1 in the MB-231 breast cancer cell line in a time-dependent manner, and expression of CDKI p21^WAF1/CIP1^ was also upregulated in a time-dependent manner. In addition, apigenin promotes acetylation of histone H3 and thus transcription of p21^WAF1/CIP1^. A nude mouse model experiment using xenotransplantation also supported this conclusion (141). This study revealed for the first time the epigenetic targeting mechanisms and effects of apigenin on breast cancer.

Paredes-Gonzalez et al. evaluated epigenetic effects of apigenin in the prevention of skin cancer. Apigenin can inhibit expression of DNMT1, 3a, 3b, and HDAC in mouse skin epidermal JB6 P+ cells, demethylate 15 CpG sites, and silence Nrf2 to promote antioxidation and prevent skin cancer (142).

Apigenin can play the role of an HDACi in inducing apoptosis in prostate cancer cells. In prostate cancer cells and xenotransplantation models, apigenin decreases expression of apoptotic protein inhibitors such as XIAP, survivin, c-IAP1, and c-IAP2, inhibits HDAC1, upregulates acetylation of Ku70, destroys the Ku70-Bax interaction, and mediates apoptosis of prostate cancer cells by separating Bax and Ku70 (143). Similar anti-HDAC effects of apigenin were also reported by a different study on prostate cancer. Apigenin (20–40 μM) inhibited HDAC activity in human prostate cancer cell lines PC-3 and 22Rv1 and reduced expression of HDAC1 and 3. Histone acetylation was also increased by apigenin-mediated downregulation of HDAC, while the cell cycle regulatory protein p21/waf1 and apoptosis protein Bax were upregulated at the transcriptional level. As shown in a mouse model, bcl2 was downregulated and Bax was upregulated in animals treated with apigenin, and growth of transplanted tumors was inhibited (144).

#### Luteolin

Luteolin is a natural flavone extracted from plants and is commonly found in traditional Chinese herbal medicines such as Lonicerae Japonicae Flos, Schizonepetae Herba, Chrysanthemi Flos and in vegetables such as broccoli, carrots, and celery. Luteolin exerts various pharmacological activities such as anti-inflammatory, anti-allergy, antitumor, anti-aging, antibacterial, antiviral, heart-protective, neuroprotective, and uric acid-reducing effects and is used to treat diabetes and Alzheimer’s disease (145–147).

Selvi et al. confirmed that luteolin regulates epigenetics multidimensionally and inhibits growth of head and neck squamous cell carcinoma cells. Treatment of mice with luteolin inhibited p300 acetyltransferase, upregulated miR-195/215 and let7C, induced by p53, downregulated miR-135a, and significantly decreased tumor growth within four weeks (148).

Kanwal et al. found that flavonoids such as luteolin, apigenin, and chrysin can inhibit DNA methylation and trimethylation of lysine 27 on histone H3, and luteolin showed the strongest inhibitory effects on DNMT. Different concentrations of luteolin (10 μM and 20 μM) inhibited DNMT in a dose-dependent manner, and molecular docking showed that luteolin can interact with DNMT residues through six hydrogen bonds. Luteolin can inhibit H3K27me3 and downregulate the expression of EZH2 in human prostate cancer DU145 cells in a concentration-dependent manner (149).

Ganai et al. found that luteolin can stably bind to class-I HDAC isomers. Luteolin, as a natural product, may have better anticancer application prospects than synthetic HDACi’s which are associated with more side effects (150).

Luteolin has been reported in many studies to be a regulator of epigenetic effects in various cancer signaling pathways. Zuo et al. found that luteolin can inhibit DNMTs and HDACs in HCT116 and HT29 colon cancer cells and promote Nrf2 demethylation and upregulate its expression, which activates the Nrf2/antioxidant responsive element(ARE) pathway and inhibits cancer cell proliferation (151). Their results show that luteolin positively regulates the epigenetic effects of anticancer signaling pathways. A study on the effects of luteolin on prostate cancer cells showed that it changed the acetylation state of the gene promoter histone, downregulated expression of 22 key genes of the cell cycle pathway such as cyclin A2 and cyclin E2, and upregulated expression of cyclin-dependent kinase inhibitor 1B (152).

Luteolin also alters histone H4 acetylation levels and regulates estrogen levels and cell proliferation in breast cancer cells. The estrogen signal pathway gene and the cell cycle pathway gene in MCF-7 breast cancer cells are regulated by this natural compound, and PLK-1 histone H4 acetylation modification which regulates the cell cycle is inhibited (153). This shows that luteolin regulates gene expression through epigenetic properties involving modified histone acetylation, which is consistent with the anti-estrogenic and cell proliferation activities of luteolin.

Luteolin, a natural inhibitor of EGFR tyrosine kinase, is also used in the development of targeted therapies. Markaverich et al. found that epigenetic regulation effects of luteolin on the epidermal growth factor signaling pathway may involve upregulation of c-Fos and p21, caused by histone H4 binding in PC-3 human prostate cancer cells (154).

#### Tangeretin

Similar to naringenin and diosmin, tangeretin is a phytochemical compound derived from citrus fruits, and it shows good antifungal, anti-inflammatory, antioxidant, and anticancer effects *in vivo* and *in vitro*. Tangeretin can inhibit tumor progression through various signaling pathways and epigenetic mechanisms (155–157).

Wei et al. examined antitumor and epigenetic activities of tangeretin (PMF1) and its derivative 4’-didemethyltangeretin (PMF2). In human prostate cancer LNCaP cells, both compounds inhibited cell growth, but PMF2 exerted stronger effects and less cytotoxicity to normal cells. PMF2 also induced apoptosis by increasing expression of proapoptotic proteins Bad and Bax, downregulating antiapoptotic proteins Bcl2, and activating caspase-3 and PARP. This study also confirmed epigenetic regulation of PMF2. PMF2 can demethylate the p21 promoter and inhibit expression of DNMT3B, HDAC1, HDAC2, and HDAC4\5\6. In addition, PMF2 can interact with DNMTs *in vitro*, which may lead to a decrease in DNMT catalytic activity (158).

#### Chrysin

Chrysin is a flavonoid extracted from *Artemisia mandshurica*, and it is also abundant in propolis, honey, and passion fruit. It exerts a wide range of pharmacological activities such as antioxidative, antitumor, antiviral, antihypertensive, antidiabetic, antibacterial, and anti-allergy effects. Due to its wide distribution in plants and low toxicity, it has become an important substrate for drug discovery (159).

Sun et al. examined anticancer effects of chrysin extracted from Chinese propolis. When breast cancer MDA-MB-231 cells were treated with chrysin, tumor cell growth and proliferation were inhibited. In a xenotransplantation model, oral administration of chrysin to immunodeficient mice reduced the size and weight of tumors. Concurrent enzyme activity analyses showed that chrysin, an HDACi, may inhibit HDAC8 activity. This study confirmed that chrysin has anti-HDAC8 activity and can inhibit chromatin remodeling in breast cancer cells to exert anticancer effects (160). Sun et al. proposed that the anti-HDAC activity of chrysin may be related to its anticancer effects (160), which was confirmed by other experiments (161).

For instance, the anti-melanoma activity of chrysin was found to be related to its anti-HDAC activity. After melanoma A375 cells were treated with chrysin, cell proliferation was inhibited, the cell cycle was blocked at the G1 phase, HDAC-2, 3, and 8 were downregulated at the translation level, and the degree of H3me2K9 methylation was decreased. In addition, chrysin upregulates expression of apoptosis-related protein p21 through protein methylation and hyperacetylation, which is considered an important factor leading to melanoma cell apoptosis (161).

TET1, which is involved in the demethylation of 5-methylcytosine, is a promising target for gastric cancer therapy, and chrysin can promote its expression to inhibit tumor progression. In gastric cancer MKN45 cells treated with chrysin, expression of TET1 and 5hmC increased, and the increase in TET1 levels promoted apoptosis and inhibited migration and invasion of gastric cancer cells. Furthermore, a CRISPR/Cas9 system was used to knock out TET1 to promote tumor growth, which showed that TET1 expression is closely associated with tumor growth, thus chrysin is expected to become an effective target for this emerging gastric cancer (162).

### Isoflavones

#### Daidzein and Genistein

Isoflavone is a flavonoid that mainly occurs in legumes. Due to similar characteristics regarding molecular structure and biological activities, isoflavones are also known as phytoestrogens. In addition to producing estrogens and antiestrogens, participating in lowering blood sugar, inhibiting obesity and type-2 diabetes, controlling inflammation, and protecting nerves, isoflavones can also be used to treat tumors related to hormone disorders, cardiovascular diseases, and human reproductive functions (163–166). The representative compounds are daidzein and genistein, which are often discussed together because of their similar origin and function.

It has been reported that isoflavones are epigenetic modulators of the Wnt pathway involved in tumorigenesis and epithelial mesenchymal transformation (EMT) intervention (167–169). In a study on colon cancer, genistein affected demethylation, upregulated the Wnt antagonist gene SFRP2, and inhibited Wnt signal transduction. Methylation-specific-PCR analysis showed that the demethylation degree of SFRP2 was up to 50% (168). In a different experiment involving the Wnt pathway, genistein led to demethylation of the CpG island of Wnt5a, a Wnt antagonist, and induction of re-expression of Wnt5a to inhibit proliferation of colon cancer cells (169); notably, these changes facilitated Wnt5a-associated demethylation regulation in early tumor cells, which may be a method for early intervention in colon cancer.

Epigenetic regulation of Wnt by isoflavones has also been demonstrated *in vitro*. Zhang et al. found that genistein included in the diet of rats can affect Wnt gene expression through DNA methylation regulation and histone modification and maintain normal Wnt signal transduction in rat colonic epithelium after exposure to the carcinogen azomethane (167), prompting the preventive effect of genistein supplementation through food intake on cancer.

The DNA methylation regulation activity of soybean isoflavones is an important mechanism for its anti-prostate cancer. Genistein and daidzein can down-regulate the methylation level of tumor suppressor gene promoter in human prostate cancer cells. When VARDI and his colleagues treated cancer cells with genistein and daidzein, EPHB2 and GSTP1 methylation was down-regulated, while BRCA1 methylation was not affected (170). Immunohistochemical results showed that the expression of BRCA1 and RASSF1A did not change, while the expression of GSTP1 and EPHB2 increased. In a different study on prostate cancer, daidzein and genistein inhibited cancer cell growth and promoted apoptosis by altering the methylation status of several genes involved in the NF-κB and p53 pathways in prostate cancer cells (171).

Genistein exerts a demethylation effect on tumor suppressor genes in esophageal squamous cell carcinoma cells. Approximately 2–20 μmol/L genistein inhibited DNMT, promoted activation of α-O6 methylguanine methyltransferase, and downregulated methylation of RARβ, p16INK4a and promoted their expression. Activity of DNMT was reduced in a dose-dependent manner with 20–50 μmol/L genistein, and HDAC activity was also inhibited by genistein (5–100 μmol/L) in a concentration-dependent manner. Additionally, the authors found that addition of 5-Aza-DCYD and trichostatin or SFN can enhance genistein effects through demethylation of tumor suppressor genes and inhibition of cell growth (172). However, the use of genistein alone or in combination with other drugs to reverse hypermethylation requires further research.

### Flavanones

#### Naringenin

Naringenin is a common citrus flavonoid which mainly occurs in the peel of citrus fruits such as oranges, lemons, and grapefruit. It exerts pharmacological activities such as liver-protective, antioxidative, antiviral, cardiovascular-protective, kidney-protective, antidiabetic, and anticancer effects and can be used to treat sepsis (173–175).

Curti et al. examined the regulation of miRNA (miR-17-3p and miR-25-5p) by naringenin in the process of anti-inflammation and antioxidation treatments of human colon adenocarcinoma. When Caco-2 cells were treated with racemic and enantiomeric Naringenin at subtoxic concentrations, expression of miR-17-3p and miR-25-5p decreased. The decrease of miR-17-3p is accompanied by the up-regulation of the transcription levels of two antioxidant enzymes, glutathione peroxidase 2 and manganese superoxide dismutase, which are encoded by it. however, downregulated expression of miR-25-5p was not consistent with mRNA expression of tumor necrosis factor-α and interleukin-6, which are pro-inflammatory cytokines and Encoded by miR-25-5p (176). These results suggest that Naringenin can achieve antioxidant activity by targeting the epigenetic mechanism of miRNAs, even though anti-inflammatory effects may be mediated by other mechanisms.

#### Hesperidin

Hesperidin occurs in fruits of Rutaceae plants such as bergamot, orange, and lemon, and are also the important active components of some botanical drugs such as Schizonepetae Spica, Citri Fructus Retinervus, Citri Reticulatae Pericarpium Viride. Hesperidin regulate epigenetic processes and exert anti-inflammatory, antioxidant, anti-obesity, and cardiovascular and anticancer effects. Hesperidin significantly reduced the growth of liver nodules in rats with liver cancer induced by diethylnitrosamine and significantly ameliorated liver histological damage. In addition, cytotoxicity of Hesperidin to HL60 cells was dose-dependent, with an IC_50_ of 12.5 mM, and at this concentration, it produced the maximum value of LINE-1 sequence hypomethylation (47%). Hesperidin also downregulated methylation of ALUM2 repeats, and the highest hypomethylation level of 32% was observed at a concentration of 6 mM (177). This study suggests that hesperidin, an active demethylation drug, can play a significant role in the treatment of liver cancer.

#### Phlorizin

Phlorizin/phloridzin is a dihydrochalcone flavonoid that mainly occurs in Rubi Fructus, and fruits, leaves, and roots of apple trees, and it has been studied for many years to examine antioxidant, anti-inflammatory, and antitumor effects on mammalian cells (178).

Sandhya et al. found that in HepG2 human liver cancer cells, MDAMB human breast cancer cells, and THP-1 leukemia cells, phlorizin and its derivatives can significantly inhibit proliferation of tumor cells, and the antiproliferative activity of fatty esters of phloridzin (a phlorizin derivative) is similar to that of sorafenib and other chemotherapeutic drugs. Furthermore, Sandhya et al. explored the antitumor mechanism of several drugs and found that fatty esters of phloridzin can inhibit DNA topoisomerase IIa activity, downregulate growth factor receptors such as the antiapoptotic genes BCL2 and PDGFR, and block the cell cycle. In addition, a variety of HDACs (HDAC1, 4, 6, 7, and 11) are downregulated by phlorizin derivatives (179). This study suggests that the chemotherapeutic effect of this phlorizin derivative may be associated with downregulation of DNA topoisomerase IIa activity, cell cycle arrest, and epigenetic activity.

#### Taxifolin

Taxifolin, a natural flavonoid which is common in Cinnamomi Ramulus, Fructus Ligustri Lucidi, Viticis Fructus, milk thistle, larch, and other plants, shows cardiovascular-protective, antibacterial, and free-radical scavenging effects and has shown good anticancer activity through a variety of pathways *in vivo* and *in vitro (* 180, 181). For example, in breast cancer cells, taxifolin can inhibit cell proliferation and migration by down-regulating β-catenin to promote EMT (182). Taxifolin has also been reported to inhibit cancer cell growth in scar cancer cells by inhibiting the PI3K/Akt/mTOR pathway (183).

Epigenetic effects of taxifolin have also been reported. After treatment with taxifolin, colony formation of mouse epidermal JB6 P+ cells was inhibited, and Nrf2, heme oxygenase-1, and NAD(P)H quinone oxidoreductase 1 were upregulated at transcriptional and translational levels. Bisulfite genome sequencing showed that methylation levels of the Nrf2 promoter were decreased by taxifolin, and expression of DNMT and DHAC were also reduced by taxifolin (184). This suggests that taxifolin can play a role in chemoprevention by promoting the expression of Nrf2 through an epigenetic pathway.

#### Silibinin

Silibinin, a representative flavanone compound, is a bioactive substance extracted from dried fruit of thistle which belongs to the family Compositae. It shows strong antioxidant functions and liver-protective, free-radical scavenging, and anti-aging, effects, among others, and it is commonly used in medicine, health care, food, and other applications.

Silibinin exerts considerable epigenetic effects and can be administered in combination with other epigenetic drugs. Mateen et al. reported that silibinin combined with an HDACi modified epigenetics and inhibited growth of non-small cell lung cancer. In vitro experiments showed that after administration of silibinin to tumor cells, acetylation of histones H3 and H4 was upregulated, accompanied by a decrease in HDAC activity, and expression of HDAC1, 2, and 3 were downregulated. After using several HDACi’s and silibinin in combination, p21 acetylation was upregulated, cytotoxicity was increased, and cell cycle arrest and apoptosis were induced (185). In vivo experiments also revealed similar epigenetic effects of silibinin, and growth of transplanted tumors was inhibited. Similarly, in a different study on non-small cell lung cancer, silibinin combined with an HDACi or DNMTi restored E-cadherin expression and reduced tumor cell migration and invasion (186).

A previous study claimed that silibinin exerts substantial preclinical anticancer activity and that it is an epigenetic regulator of prostate cancer. When human prostate cancer cell lines DU145 and PC3 were treated with silibinin, expression of EZH2, SUZ12, and EED was downregulated, accompanied by an upregulation of H3K27me3 methylation. In addition, silibinin increased the activity of DNMT and suppressed the expression of HDAC1-2 (187). Silibinin also played an epigenetic targeting role in the process of antiproliferation of bladder cancer. Silibinin showed considerable cytotoxicity to bladder cancer cells with different TP53 statuses. In the highest grade tumors (TP53 mutation status is level 3), silibinin induces global DNA hypomethylation. In low-grade tumor cells (wild type TP53 gene), silibinin downregulated expression of HDAC and HAT (188).

Like many flavonoids, silibinin also regulates noncoding RNAs. In the exploration of anti-breast cancer, Silibinin has been found to induce apoptosis by regulating microRNA. When MCF-7 cells were treated with silibinin, decreased expression of miR-21 and miR-155 was detected, and apoptosis occurred in a dose- and time-dependent manner. Shengxin analysis was used to predict the potential targets of two kinds of microRNAs, caspase-9 and bid. PCR results showed that expression of caspase-9 and bid were increased, which was consistent with the results of the dry and wet experiments (189).

### Flavanols

#### EGCG

Tea is the second most commonly consumed beverage in the world after water and is particularly common in Asia. Catechins are flavanols, and they are the main functional components of tea, comprising various monomer structures among which EGCG is most prominent due to its regulatory effects on many aspects of human physiological and pathological processes. EGCG has neurotrophic, anticancer, and antifibrotic pharmacological activities and plays an important role in inhibiting oxidation and inflammation (190–192). Epigenetic regulation of cancer by EGCG alone or in combination with other compounds was described in many recent studies.

Sheng et al. examined methylation regulation of EGCG in breast cancer. The tumor suppressor gene SCUBE2 is frequently methylated and silenced in breast cancer, while EGCG has been reported to regulate the levels of methylation in the promoter region of various tumor-related genes. In this study, MDA-MB-231 and MCF-7 cell lines were treated with EGCG, and an MTT assay showed that growth of breast cancer cells was inhibited and the cell viability was significantly decreased; migration and invasion abilities of breast cancer cells were also inhibited. Regarding the underlying mechanism, EGCG reactivated SCUBE2 gene expression. Real-time PCR revealed that transcription of DNMT1, DNMT3a, and DNMT3b was downregulated, and the degree of DNA methylation was also reduced (15).

EGCG has been reported to reverse both DNA methylation and histone acetylation abnormalities to induce apoptosis in breast cancer cells and inhibit cancer development. When EGCG and sulforaphane (SFN) were used in a breast cancer-transformed cell system, cell viability decreased and apoptosis and cell cycle arrest were induced. EGCG decreased the activities of DNMT1 and HDAC1, and combined administration was more effective in regulating epigenetics. To further explore the regulation of epigenetic aberrations, histone H3 acetylation and DNA methylation levels were examined, and cotreatment with EGCG and SFN significantly promoted global acetylation of histone H3 in the breast cancer-transformed cell system. Cluster analysis and screening of methylation status using probes showed that the methylation status of tumor-related genes was changed to varying degrees. In addition, combined treatment inhibited oncogene Septin9 and promoted expression of the tumor suppressor gene DCBLD2. EGCG and/or SFN inhibited xenograft tumor growth in breast cancer mice, and the combined effect of the two drugs was stronger (193). This new dietary combination shows promising prospects for the prevention and treatment of breast cancer.

Deb et al. conducted experiments and proposed that green tea polyphenol (GTP) and its main active ingredient EGCG can remedy epigenetic disorders caused by abnormal expression of matrix metalloproteinase-3 (TIMP-3) in prostate cancer. GTP/EGCG decreased invasiveness and migration of prostate cancer cells and decreased the activity of matrix metalloproteinases (MMPs) such as MMP-2 and MMP-9. TIMP-3 was upregulated at a transcriptional and translational level, whereas acetylation of class-I HDAC, EZH2, and H3K9/18 and H3K27 trimethylation were downregulated; low expression of class-I HDAC and EZH2 was due to activation of TIMP-3. Deb et al. then used clinical samples for verification. After analyzing data of patients treated with EGCG, they found decreased expression of MMP-2, MMP-9, class-I HDAC, and EZH2, and TIMP-3 was upregulated, which was consistent with experimental results at a cellular level (16).

Zhao et al. reported that EGCG can regulate lncRNAs and miRNAs to reverse gastric cancer. When gastric cancer AGS and SGC7901 cell lines were treated with different doses of EGCG, growth of tumor cells was inhibited in a time- and dose-dependent manner. Bioinformatics showed that EGCG altered gene expression in gastric cancer cells and participated in the regulation of cell metastasis. Expression of LINC00511, which promotes tumor metastasis and proliferation, can be downregulated by EGCG, and miR-29b, as a downstream target of LINC00511, can be inhibited by this lncRNA, thus increasing expression of carcinogenesis-related KDM2A (a kind of KDM) (17). This study reflects epigenetic effects of EGCG on proliferation, metastasis, and invasion of gastric cancer, particularly regarding targeting of the LINC00511/miR-29b/KDM2A axis.

Multilevel epigenetic effects of EGCG on hematological tumors have also been examined. During acute promyelocytic leukemia (APL), GTP has been shown to inhibit cell proliferation and cause tumor cell apoptosis. Borutinskaitė et al. proposed that during the antitumor process, EGCG regulates expression of cell cycle-related genes, promotes expression of transcriptional regulatory protein families C/EBPα and C/EBPϵ, and upregulates PCAF and p27. RT-qPCR results showed that DNMT1, HDAC1, and HDAC2 were inhibited by EGCG, and G9A, which is related to H3K9me2 methylation, was also downregulated. Expression of genes related to PRC2 with HMT activity was inhibited at the protein level, and the binding effect of these genes with acetylated histone H4 and acetylated H3K14 is reduced, while in cell cycle-related genes such as C/EBPα and acetylated H3K14, the relationship between H4 and H4 has become closer (194). EGCG is not only used as a cell cycle blocker but also has an epigenetic remedy, and it has considerable potential for application in the treatment of APL.

#### Theaflavins

EGCG is a representative biologically active constituent of green tea, and theaflavins are natural flavonoids which are highly abundant in black tea where they are responsible for color and taste; moreover, these compounds are considered effective antioxidant and anticancer agents (195–197).In an enzyme inhibition test, theaflavins were identified as natural DNMT inhibitors. it inhibits DNMT1 by 65%, and the IC_50_ value of DNMT1 inhibition was 85.33 µM (198).

A different study found that theaflavins can reduce the activity of DNMT1 and DNMT3a and inhibit the proliferation of colon cancer cells HCT-116 and the progression of solid tumors induced by EAC in mice. The authors found that theaflavins can downregulate the activity of DNMT, both *in vivo* and *in vitro*. In addition, immunohistochemical analysis of mouse tumor tissues showed that theaflavins inhibited the expression of DNMT (199). This study demonstrates that theaflavins act as potential DNMT inhibitors in colorectal cancer.

### Anthocyanidins

Anthocyanidins are natural water-soluble pigments belonging to the group of flavonols which are commonly found in plant petals and fruits. Because of their active biological activity and coloring function, they are commonly used in the production of food, drinks, and medicine. These flavonoids exert beneficial functions including vision-protective, antioxidative, antitumor, and anti-cardiovascular disease effects, and they can be used for preventing and treating type-2 diabetes and treating obesity (200–202). Due to the similarity of source and structure, different types of anthocyanidins show similar epigenetic effects in function.

In a report on the prevention of skin cancer, anthocyanidins were shown to modulate epigenetic activation of the NRF2-ARE pathway and inhibit carcinogenesis in JB6 P+ cells. Kuo et al. treated JB6 P+ cells with the anthocyanidin delphinidin and found that ARE-driven luciferase activity was markedly upregulated, Nrf2 and its downstream antioxidant and carcinogenic detoxion-related genes were upregulated, and Nrf2-ARE was activated. Kuo et al. further investigated epigenetic activity of anthocyanidins during skin cancer. Delphinidin not only demethylates the Nrf2 promoter, but also inhibits expression of DNMTs and HDACs (203).

A different study on skin cancer study discussed the epigenetic regulation of Nrf2-ARE by pelargonidin, which is a commonly studied anthocyanidin. Similar to delphinidin, pelargonidin promoted activation of the Nrf2-ARE signaling pathway, upregulated expression of the Nrf2 target gene, demethylated the Nrf2 promoter at an epigenetic level, and inhibited expression of DNMT and HDAC (204).

The activity of anthocyanidins that target miRNAs has also been examined. Black raspberry anthocyanins (BRB) inhibit growth of human colon cancer cells by upregulating miR-24-1-5p. BRB upregulated the expression of miR-24-1-5p in a human colorectal cancer cell line and downregulated β-catenin in an AOM/DSS-induced mouse model. In addition, the authors used bioinformatics to predict β-catenin as a target gene of miR-24-1-5p and confirmed this using RT-qPCR and western blotting. The results showed that miR-24-1-5p inhibited the expression of β-catenin. In cell experiments, miR-24-1-5p regulated the downstream target genes of the Wnt/β-catenin signaling pathway, and cyclinD1, c-Myc, and CDK4 were downregulated at the transcriptional and translational level (205). This study suggests that miR-24-1-5p may act as a regulator of the Wnt/β-catenin signaling pathway to inhibit colorectal cancer, which is one of the possible mechanisms of anthocyanin anticancer effects through epigenetic pathways.

## Discussion

### Epigenetics as a Link Between Flavonoids and Prevention and Treatment of Cancer

**Figure 4:**
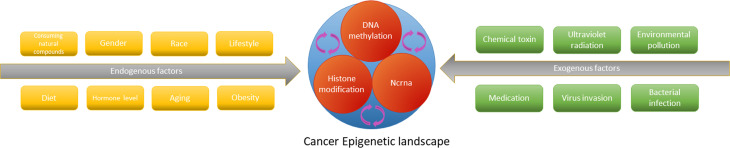
The epigenetic cascade network mediated by endogenous and exogenous factors interferes with tumorigenesis and progression by affecting gene expression.

The epigenetic genome is affected by acquired factors, and natural compounds consumed by dietary and medicinal plants are involved in shaping the dynamic balance of epigenetic state (206, 207) (**Figure 4**). Natural compounds derived from natural medicines and foods which may act as epigenetic regulators to remedy adverse gene expression patterns have been accepted by the scientific community. Flavonoids are indispensable compounds in in human body, and their epigenetic effects may help counteract a variety of diseases.

Flavonoids show activity against various modifications in the multilevel epigenetic interaction network of cancer, from DNA histones to noncoding RNAs. Specifically, one kind of flavonoids can be involved in the remodeling of a variety of related enzymes or non-coding RNA in modifying one level of cancer epigenetic regulation. Also, one flavonoid compound can modify multi-level epigenetic network, playing a role in regulating DNA methylation, histone modification and regulating non-coding RNA, and at the same time up-regulate tumor suppressor genes and down-regulate oncogenes to interfere with the occurrence and progression of cancer (**Figure 1**). Remodeling of these epigenetic links eventually leads to the accumulation of changes in cell metabolism and self-renewal and ultimately results in the loss of tumor cell carcinogenicity (208–210).

Considering the heterogeneity of the population and the multi-target complexity of the epigenetic modification network, population-based clinical trials and observational studies have not yet shown sufficient clear links for flavonoids to target epigenetics. However, the success of *in vivo* and *in vitro* studies has fully demonstrated the driving force of flavonoids on epigenetics and has shown considerable therapeutic effects on different cancers (**Table 2**). This also encourages people to design more feasible solutions to search for the chain of evidence that these phytochemicals is effective in maintaining the human body’s epigenetic balance and disease prevention.

**Table 2:** Epigenetic effects of flavonoids on different types of cancers *in vitro* and *in vivo*.

Flavonoid subtype	Phytochemical	Epigenetic modifications	Molecular targets	Regulation of cell phenotypes	Dose	In vitro model	In vivo model	Cancer species	References
Flavonols	Kaempferol	HDAC inhibitor G9a inhibitor	Down-regulation of P62; Up-regulation of p-JNK and CHOP	↑Autophagy	25,50,100 µM	AGS, SNU-216, NCI-N87, SNU-638, and MKN-74 cell lines		Gastric cancer	(14)
	Kaempferol	HDAC inhibitor		↓Cell viability ↓proliferation	5-100 µM	Hepg2,Hep3B cells		Liver cancer	(109)
	Kaempferol	HDAC inhibitor		↓Cell viability ↓proliferation	5-100 µM	HCT-116 cells		Colon cancer	(109)
	Kaempferol	DNMT1 inhibitor DNMT3b inhibitor DNMT3a inhibitor	Down-regulation of DACT2 and β-catenin	↓Proliferation ↓Migration	1.25-150 μM; 75 and 150 mg/kg	HCT116,HT29 and YB5 cell lines	Nude mice	Colorectal cancer	(110)
	Quercetin	Promotion of mir-146a expression	Activation of caspase-3 and Bax; Inhibition of EGFR expression	↑Apoptosis ↓Proliferation ↓Invasiveness of tumor cells	0,25,50,80 and 100 µm/ml; 10 mg/kg	MCF-7 and MDA-MB-231 cells	Athymic nude mice	Breast cancer	(129)
	Quercetin	Promotion of mir-217 expression	Down-regulation of kras	↓Viability	0, 5, 10, 50, and 100 μM	143B cells		Osteosarcoma	(130)
	Quercetin	Promotion of mir-200b-3p expression	Down-regulation of Notch signaling pathway	↑Asymmetric cell division ↓Self-renewal ↓Proliferation	50 μm	Aspc1 and PANC1 cells		Pancreatic cancer	(127)
	Quercetin	Promotion of mir-34a expression	Down-regulation of SIRT1; Up-regulation of p53 and p21	↑Apoptosis ↓Proliferation	31.25 μM	Hepg2 cells		Liver cancer	(128)
Flavones	Diosmin	DNA hypermethylation		↑Apoptosis ↓Proliferation	0,50,100,150,200 and 250 μM	DU145 cells		Prostate cancer	(134)
	Apigenin	HDAC inhibitor	Down-regulation of cyclin A, B and CDK1; Up-regulation of p21^waf1/CIP1^	↓Proliferation ↓Cell cycle arrest	0, 10, 20 and 40 μM	MDA-MB-231 cells	Athymic nude mice	Breast cancer	(141)
	Apigenin	HDAC inhibitor	Decrease the expression of XIAP, survivin, c-IAP1 and c-IAP2; Disruption of Ku70–Bax interaction	↑Apoptosis ↑Cell cycle block ↓Cell survival rate	5–40 μM; 20,50 μg	PC-3 And DU145 cells	Athymic nude mice	Prostate cancer	(143)
	Apigenin	HDAC1 inhibitor HDAC3 inhibitor	Up-regulation of p21^waf1/CIP1^ and bax in transcriptional levels	↑Growth Arrest ↑Apoptosis	20-40 μM; 20 and 50 μg	PC-3 and 22Rv1 cells	Athymic nude mice	Prostate cancer	(144)
	Luteolin	Inhibition of p300 acetyltransferase and histone acetylation; Up-regulation of mir-195/215,let7c; Down-regulation of mir-135a	Up-regulation of POFUT and DICER; Down-regulation of E2F2,DOK2	↑Cell cycle arrest ↓Cell migration	10µM and 25µM; 100 mg/kg	KB cells	Xenograft mice	Head and neck squamous cell carcinoma	(148)
	Luteolin	DNMT inhibitor HMT inhibitor Down-regulation of EZH2			10- μM,20- μM	DU145 cells		Prostate cancer	(149)
	Luteolin	DNMT inhibitor HDAC inhibitor	Up-regulation of Nrf2,HO-1, and NQO1 expression	↓Cell proliferation ↓Cellular transformation	10- μM and 20- μM	HCT116 And HT29		Colon cancer	(151)
	Chrysin	HDAC8 inhibitor	Promoting p21 expression	↓Cell growth ↑Cell differentiation	10,20, and 40 μM; 45 or 90 mg/kg	MDA-MB-231 cells	Nude mice	Breast cancer	(160)
	Chrysin	HDAC2,3,8 inhibitor	Promoting STAT-1 and p21 expression	↓Cell proliferation ↑Cell cycle	40,120 μM	A375 cells		Melanoma	(161)
	Chrysin	Increase the expression of TET1 and 5hmc	Promoting the expression of BAX and inhibiting the expression of bcl2	↑Apoptosis ↓Cancer cell migration and invasion	0,10,20,40,80,160 μM; 20 mg/kg	MKN45 cells	Nude Mice Xenograft Model	Gastric cancer	(162)
Isoflavones	Genistein	Demethylation	Up-regulation of SFRP2, inhibition of Wnt signal transduction; Decreasing nuclear β-catenin and increasing phospho-β-catenin accumulation	↓Cell viability ↓Proliferation ↑Apoptosis	75 mmol/L	DLD-1 cells		Colon cancer	(168)
	Genistein	Decreasing of DNA methylation level	Up-regulation of WNT5a	↓Cell proliferation	75 μmol/l	SW1116 cells		Colon Cancer	(169)
	Genistein	DNA methylation regulation and histone modification	Down-regulation of Sfrp2,Sfrp5 and Wnt5a		140 mg/kg		Timed-pregnant Sprague-Dawley rats	Colon cancer	(167)
	Genistein	DNMT inhibitor	Up-regulation of RARβ And p16INK4a	↓Cell growth	2-100 μmol/L	KYSE 510, KYSE 150 cell lines		Esopharachaquest cell carcinoma	(172)
Flavanones	Hesperidin	Hypomethylation		↓Cell viability	0.78 to 25 mm;250,500,1000 ppm	HL60 Cell line	Pathogen-free male Sprague-Dawley rats	Liver cancer	(177)
	Silibinin	HDAC1,2,3 inhibitor		↓Migration ↓Invasion	3.75-12.5 µM	H1299 cells		Non-small cell lung cancer	(186)
	Silibinin	Improvement of total DNMT activity; Inhibition of the activity of HDAC1, 2	Down-regulation of EZH2,SUZ12 and EED		25–75 µg/ml	DU145 and PC3 cell lines		Prostate cancer	(187)
	Silibinin	Down-regulation of mir-21 and mir-155	Up-regulation of caspase-9 and bid	↓Proliferation ↑Apoptosis	0–300 µM	MCF-7 cells		Breast cancer	(189)
Flavanols	EGCG	DNMT1 inhibitor DNMT3a inhibitor DNMT3b inhibitor	Up-regulation of SCUBE2	↓Cell growth ↓Migration and invasion	0-100 μM	MDA-MB-231 and MCF-7 cell lines		Breast cancer	(15)
	EGCG	Regulation of LINC00511/mir-29b/KDM2A axis		↓Proliferation ↓Metastasis ↓Invasion	0-100 μM	AGS and SGC7901 cell lines		Gastric cancer	(17)
	EGCG	DNMT1 inhibitor HDAC1 inhibitor HDAC2 inhibitor G9a inhibitor	Up-regulation of C/EBPα,C/EBPϵ,PCAF and p27	↓Proliferation ↑Apoptosis	30, 40 μM	NB4 and HL-60 cells		APL	(194)
	Theaflavins	DNMT1 inhibitor DNMT3a inhibitor		↓Cell Viability	0,25, 50,75, 100, 125, 150 μg/ml; 0.02 mg/kg,10 mg/kg	HCT-116 cells	Swiss albino mice	Colon cancer	(199)
Anthocyanidins	Delphinidin	Dnmts inhibitor Hdacs inhibitor	Up-regulation of Nrf2 and ARE	↓Cell carcinoma	0-100 μM	JB6 P+ cells		Skin cancer	(203)
	Pelargonidin	Dnmt inhibitor Hdac inhibitor	Up-regulation of Nrf2,NQO1 and HO-1	↓Cell deterioration	0-,10,30,50 μM	JB6 P+ cells		Skin cancer	(204)

### Current Challenges for Flavonoid-Targeted Epigenetic Antitumor Research and Development

Research on flavonoids is inspired by their biological effects, including, but not limited to, antioxidation, free-radical scavenging, cell cycle regulation, anti-estrogens affects, protein tyrosine kinase inhibition, and epigenetic activity (211–214). Plant compounds may exert a plethora of effects (215), which may, however, harbor some disadvantages (216, 217). Flavonoids produce anticancer effects through various mechanisms, and unspecific action may be problematic for potential drug administration. This is also a clinically important consideration regarding the use of phytochemicals (213, 218). In addition, the hierarchical interaction and reversibility of the epigenetic network suggests that when flavonoids modulate epigenetic modifications in multiple dimensions, they may cause deviations in efficacy and expected results, This is also a problem faced by other epigenetic drugs in the application (219, 220). However, based on a large number of experimental studies, we believe that flavonoids have beneficial preclinical benefits of regulating cancer epigenetics. Their broad biological activities, however, should not be a reason to dismiss flavonoids from disease research and treatment; instead, the underlying mechanisms warrant further research to exploit the inherent therapeutic potential.

Even though clinical flavonoid research focusing on tumor epigenetics has made considerable progress recently, current evidence for clinical application is still insufficient (218). A series of problems such as insufficient bioavailability currently prevent clinical application of various plant products including flavonoids. Fortunately, the academic community has begun to tackle this problem and has provided effective solutions such as combined administration with other plant compounds or anticancer drugs to improve anticancer efficacy and nano-drug carrier system to improve the pharmacokinetic characteristics of flavonoids. In addition, considering that dosages used in cell culture and animal models may not be physiological and may thus not be transferable to humans, determining the most effective dosage of flavonoids is a crucial part of basic and clinical research to effectively and safely exploit epigenetic antitumor effects of flavonoids (221).

## Conclusions and Future Prospects

Dysfunctional epigenetics have been confirmed in various tumors in numerous studies. Flavonoids are important epigenetic regulators and are commonly consumed worldwide. In vivo and *in vitro* studies confirm that flavonoids can inhibit cancer by restoring dysfunctional epigenetics and intervening in DNA methylation, histone modification, and expression of noncoding RNA. Whether used alone or as an adjuvant therapy, flavonoids have shown significant efficacy. Despite many challenges, the application prospects of flavonoids are promising. Future research will provide further insights into the targeted epigenetic dimensional adjustment of flavonoids. More clinical trials are required to elucidate effects on cancer and the feasibility of clinical administration of flavonoids. 

## Author Contributions

The research project was designed by WJ, TX, CL, and CS. WJ collected the literature, drew structures. WJ and TX wrote the manuscript and checked the Tables and Figures as well as grammar of manuscript. CL, JL and WZ revised the manuscript. CS and WJ participated in and helped draft the manuscript. All authors contributed to the article and approved the submitted version.

## Funding

This work was supported by the National Natural Science Foundation of China (grant number 81973677).

## Conflict of Interest

The authors declare that the research was conducted in the absence of any commercial or financial relationships that could be construed as a potential conflict of interest.
